# Association between Sarcopenia and Metabolic Syndrome in Middle-Aged and Older Non-Obese Adults: A Systematic Review and Meta-Analysis

**DOI:** 10.3390/nu10030364

**Published:** 2018-03-16

**Authors:** Huaqi Zhang, Song Lin, Tianlin Gao, Feng Zhong, Jing Cai, Yongye Sun, Aiguo Ma

**Affiliations:** The College of Public Health, Qingdao University, 38 Dengzhou Road, Qingdao 266021, China; huaqi_erin@163.com (H.Z.); 15146001303@163.com (S.L.); gaotl@qdu.edu.cn (T.G.); zhfoeg@163.com (F.Z.); jingfox@163.com (J.C.); yongye.sun@126.com (Y.S.)

**Keywords:** sarcopenia, meta-analysis, metabolic syndrome, non-obese, middle-aged and older

## Abstract

The associations between sarcopenia and metabolic syndrome (MetS) in non-obese middle-aged and older adults remain controversial. Thus, this meta-analysis aimed to evaluate the overall prevalence of MetS and the correlations between sarcopenia and MetS in middle-aged and older non-obese adults. We performed a systematic searched strategy using PUBMED, EMBASE and Web of Science databases for relevant observational studies investigating sarcopenia and MetS up to 11 May 2017. The polled prevalence of MetS and odds ratios with 95% confidence intervals (CI), as well as subgroup analyses were calculated using a random effects model. Twelve articles with a total of 35,581 participants were included. The overall prevalence of MetS was 36.45% (95% CI, 28.28–45.48%) in middle-aged and older non-obese adults with sarcopenia. Our analysis demonstrated a positive association between sarcopenia and MetS (OR = 2.01, 95% CI, 1.63–2.47). The subgroup analysis showed that both larger cohort size and the use of dual-energy X-ray absorptiometry to measure body composition can enhance the relationship. Our study revealed that a higher proportion of MetS in middle-aged and older non-obese people with sarcopenia. Moreover, sarcopenia was positively associated with MetS in this population. Further large-scale prospective cohort studies are needed to investigate the causality between sarcopenia and MetS.

## 1. Introduction

Sarcopenia is defined as the loss of muscle mass, strength and decline in physical performance with age. The European Working Group on Sarcopenia in Older People (EWGSOP), the International Working Group on Sarcopeina and the Asia Working Group for Sarcopenia (AWGS) recommend using the simultaneous presence of both low muscle mass and low muscle function to define sarcopenia, although their phenotypes and specific cut-off points for diagnosing sarcopenia are not completely uniform [1,2,3]. These criteria were further validated as good predictors of future mobility impairment, hospitalization and death [1,4]. The diagnostic techniques used to characterize sarcopenia include non-imaging diagnostic techniques (e.g., questionnaires, physical performance, general anthropometry, bioelectrical impedance analysis, serum and urinary biomarkers) and imaging techniques (e.g., whole-body dual X-ray absorptiometry, sonography, magnetic resonance imaging and computed tomography) [5]. Although evaluating the prevalence of sarcopenia has been limited by diagnostic techniques and criteria, the prevalence of sarcopenia has been reported to range from at least 4.6% in community-dwelling older people (mean age of 67 years) [6] to 25% in hospitalized older people (mean age of 82.8 years) [7]. By 2050, more than 200 million people worldwide are predicted to suffer from sarcopenia [2]. 

Metabolic syndrome (MetS) is a cluster of metabolic abnormalities (abdominal obesity, high blood pressure, high blood glucose, and blood lipid abnormalities) that is associated with an increased risk of cardiovascular morbidity and mortality, as well as all-cause mortality [8,9,10]. The prevalence of metabolic syndrome is increasing worldwide. The unadjusted MetS prevalence was 34.1% among US adults (aged ≥ 20 years) in the National Health and Nutrition Examination Survey 1999–2006. Similarly, according to data from the Korean National Health and Nutrition Examination Survey 1998–2007, the crude MetS prevalence was 31.3% in Korean adults (mean age 49.9 years) in 2007 [11]. However, these results did not report MetS prevalence by different body mass index (BMI) categories.

The majority of previous studies on MetS have focused on whole populations or obese subpopulations. Recently, several studies have addressed the high prevalence and increased risk of MetS in individuals who have BMI in the upper normal-weight and slightly overweight range [12]. As a geriatric and aging disease, sarcopenia is known to be associated with various metabolic disorders, including obesity [13], insulin resistance [14], diabetes [15], dyslipidemia [16], and hypertension [17]. Several studies have revealed a relationship between MetS and sarcopenia [18,19,20]. It is worth noting that sarcopenia showed an association with MetS in older people with normal BMI and may be an early predictor for MetS susceptibility in non-obese older adults [20]. The potential mechanisms linking MetS with sarcopenia are as follows: as a primary site for glucose and fatty acid metabolism, muscle mass loss promotes insulin resistance, which leads to MetS and type 2 diabetes; secreted proteins or myokines from skeletal muscle fibers (especially type IIb muscle fibers) have been shown to reverse the pathological consequences of metabolic disorders by counteracting the metabolic effect of adipokines secreted by adipose tissue, and these muscle fibers are selectively lost during the aging process [21]. 

## 2. Methods

This systematic review and meta-analysis was conducted and reported according to the Preferred Reporting Items for Systematic Reviews and Meta-Analysis (PRISMA) statement (PRISMA Checklist, Table S1) [22].

### 2.1. Literature Search 

Two authors (S.L. and H.Z.) independently searched published studies indexed in PUBMED, EMBASE and Web of Science databases from inception to 11 May 2017. The keywords used in search were those referring to metabolic syndrome (metabolic syndrome X, metabolic syndrome, Plurimetabolic syndrome, Reaven syndrome, syndrome X, dysmetabolic syndrome X, cardiovascular syndrome and insulin resistance syndrome) and sarcopenia (sarcopenia, sarcopenic obesity, loss of skeletal muscle, loss of muscle mass and strength, and muscle wasting). Conference abstracts were also included, and authors were contacted to obtain missing information when necessary. References of selected retrieved articles were also manually reviewed.

### 2.2. Inclusion and Exclusion Criteria 

We included published quantitative studies that examined the association between MetS and sarcopenia and/or reported the prevalence of MetS in middle-aged and older non-obese sarcopenic adults with a mean age ≥40 years, where non-obese and obese was divided by BMI at the design stage or the relationship between MetS and sarcopenia in the general population was assessed according to normalized or adjusted for BMI. We only included studies that calculated BMI by the standard formula: weight (kg) divided by the square of height (m).

We included studies that calculated skeletal muscle mass index (SMI) for each participant using any of the following formulas: (1) the appendicular skeletal muscle mass (ASM), which is the sum of lean mass in the upper and lower limbs measured by dual energy X-ray absorptiometry (DXA) scans or bioelectric impedance analysis (BIA) (kg), was divided by the weight (kg) (ASM/WT); (2) ASM was divided by the square of height (m) (ASM/Ht^2^); (3) ASM was divided by BMI (kg/m^2^) (ASM/BMI); (4) ASM was divided by height squared (m^2^) and then divided by weight (kg) ((ASM/Ht^2^)/WT). We also included studies that defined sarcopenia using handgrip strength (kg) alone or combined with SMI and gait speed (m/s), as well as by the 24-h urinary creatinine method.

We included studies that diagnosed MetS according to the criteria of the American Heart Association, the National Heart, Lung, and Blood Institute, and the criteria of the International Diabetes Federation (AHA/NHLBI/IDF) or according to the criteria established by the National Cholesterol Education Program Adult Treatment Panel III (NCEP-ATPIII), as well as the criteria of MetS commonly used in Japan.

We excluded studies in which we were unable to extract data of non-obese individuals from the obese or could not calculate a standardized effect size (e.g., when data were represented in *T*-scores or *z*-scores), as well as studies without a clear definition or diagnostic criteria for MetS or sarcopenia (e.g., metabolic phenotype, dynapenia or lean mass loss). We also excluded studies that were conducted on clinical patients but not community samples to avoid bias.

### 2.3. Study Selection and Data Extraction

Two authors (T.G. and F.Z.) independently reviewed titles and abstracts of all citations. After all abstracts were reviewed, the short-listed articles of interest were compared between the two investigators and evaluated with the inclusion and exclusion criteria listed previously. Disputed studies were resolved by consensus with a third author (A.M.). 

The following information was extracted: surname of the first author, year of publication, country in which the study was performed, characteristics of the study population (e.g., cohort size and demographics), diagnostic criteria for MetS and sarcopenia, and adjustment factors. All information was recorded on a standardized data collection form and was checked by all authors. 

### 2.4. Quality Assessment

The quality of each study was independently evaluated by two authors (J.C. and Y.S.) using the Newcastle–Ottawa Scale for cohort, case-control studies in three aspects, including the selection of study participants, the comparability of the groups and assessment of outcome measures [23] . Cross-sectional studies were evaluated by the modified Newcastle-Ottawa Scale [24]. Discrepant opinions between authors were resolved by consensus. Studies with a Newcastle-Ottawa scale score ≥ 5 were regarded as high quality. 

### 2.5. Statistical Analysis

Pooled mean effect size was calculated using meta-analysis software Stata V.11.0 (Stata Corp, College Station, TX, USA) and R. The random-effect model was used as it assumes varying effect sizes between studies, because of differing study designs and populations. We used reported prevalence, odds ratio (OR), converted prevalence and converted OR from the published data. Both the prevalence of MetS together with 95% confidence intervals (CIs) and the OR with 95% CI of MetS among middle-aged and older adults with and without sarcopenia were calculated. For studies with several estimates, we extracted those reflecting the most representative non-obese population. We conducted subgroup analyses to investigate the potential differences and heterogeneity according to adjustment or not, continent, and diagnosis of MetS and sarcopenia. The statistical significance level was set at *p* < 0.05 unless otherwise specified. Sensitivity analysis was performed to evaluate the robustness of results, in which pooled estimates were calculated after removing one study in each turn. Study heterogeneity was measured using the Cochran’s Q and I-squared statistics, assuming that a *p* ≤ 0.10 for the former and a value ≥ 50% for the latter indicated a significant and substantial heterogeneity. Publication bias was assessed using funnel plot, Egger’s regression test and the trim and fill methods. 

## 3. Results

### 3.1. Literature Search

**Figure 1:**
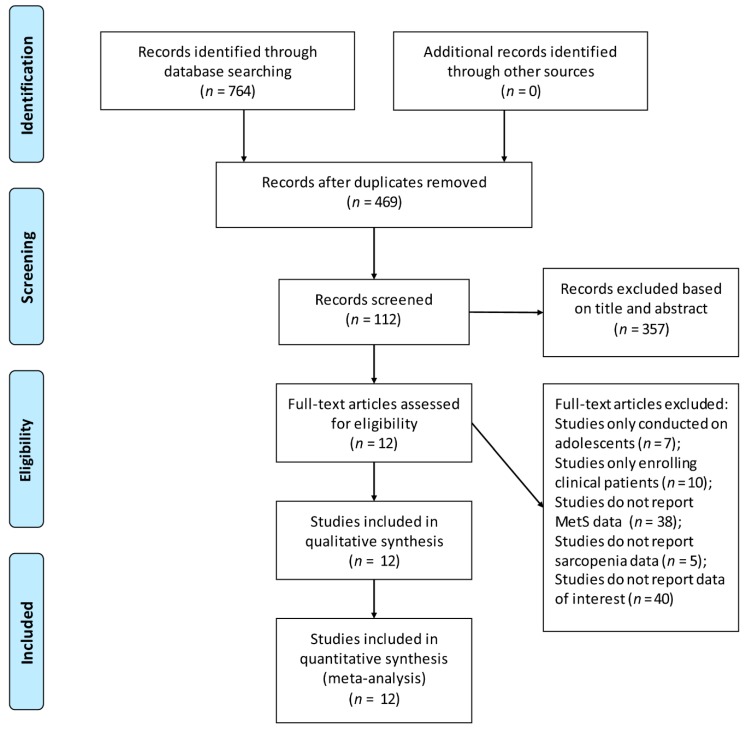
Flow diagram of the literature search.

We initially identified 764 potential articles. After removing duplicates and reviewing the titles and abstracts, 652 articles were removed. Then, 112 articles underwent full-text review according to the eligibility criteria, which excluded 100 articles. In total, 12 articles were retrieved in our final meta-analysis [18,20,25,26,27,28,29,30,31,32,33,34]. Among the 12 included articles, ten provided available data for evaluating the prevalence of MetS in sarcopenic adults and there were 13 studies assessing the association between MetS and sarcopenia. All these studies provided only cross-sectional data. The selection process is schematized in Figure 1. 

### 3.2. Study and Participants’ Characteristics

Characteristics of the included studies are summarized in Table 1. All of the studies were conducted among community-dwellers. The studies investigating the prevalence of MetS included a total of 4427 non-obese sarcopenic middle-aged and older adults with mean ages of 50.9–83.1 years, among whom 65.1% were women. These participants were compared with 31,154 participants without sarcopenia with mean ages of 43.1–82.0 years, among whom 52.5% were women. Ten studies were performed in Asia, one in North America and one in Asian and Oceania. Eleven articles used NCEP-ATPIII to define MetS and one used NCEP-ATPIII. Sarcopenia was diagnosed by DXA in eight studies, while the rest used HGS, BIA/HGS/GS and 24-h urine creatinine tests.

### 3.3. The MetS Prevalence among Middle-Aged and Older Non-Obese Adults with Sarcopenia

**Figure 2:**
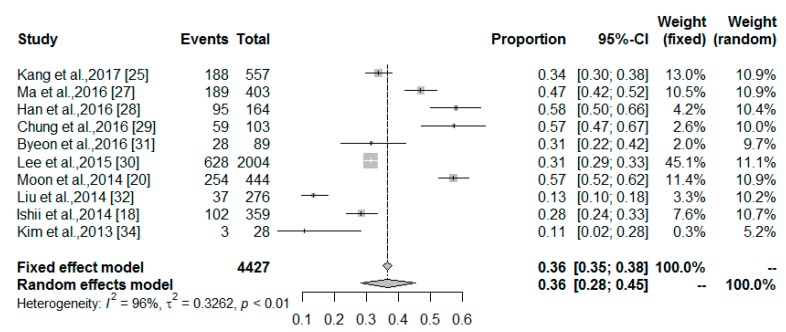
Forest plot of the meta-analysis for the overall MetS prevalence in middle-aged and older non-obese adults with sarcopenia.

Full details of the MetS prevalence among middle-aged and older non-obese adults with and without sarcopenia are summarized in Table 2. The overall MetS prevalence among the 4427 sarcopenia patients was 36.45% (95% CI: 28.28–45.48%; *I*^2^ = 96%; Figure 2), while the prevalence among the 37,045 middle-aged non-obese adults without sarcopenia was 22.81% (95% CI: 17.97–28.51%; *I*^2^ = 99%). Table 2 also shows the MetS prevalence among the non-obese sarcopenic population stratified by geographical region, the MetS definition, sarcopenia measure, and cohort size (*n* ≥ 200 versus *n* < 200). The MetS prevalence in non-obese adults with sarcopenia was higher in studies conducted in North America that used 24-h urinary creatinine analysis to diagnose sarcopenia, while the MetS prevalence was lower in studies that only used HGS and BIA/HGS/GS for diagnosis. Cohort size did not significantly affect the MetS prevalence in the sarcopenic population, although the prevalence was slightly higher in studies with cohort sizes < 200 patients. In contrast, the MetS prevalence was significantly influenced by cohort size in the population without sarcopenia. Linear regression tests of funnel plot asymmetry suggested that there were no significant publication biases in either the sarcopenia (*p* = 0.77) or non-sarcopenia (*p* = 0.53) groups. Additionally, the polled estimates were unchanged after trim and fill analyses, signifying that no additional studies are needed.

### 3.4. Odds Ratio of MetS in Middle-Aged and Older Non-Obese Adults with Sarcopenia

**Figure 3:**
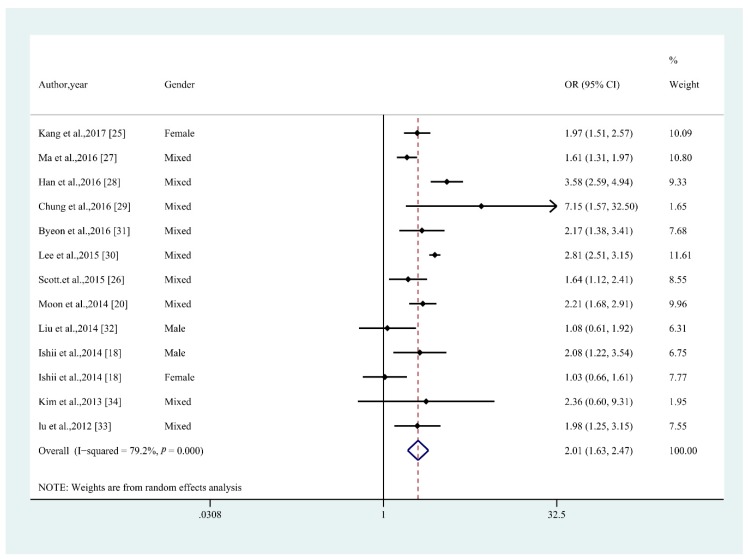
Funnel plot with pseudo 95% confidence limits for the association between MetS and sarcopenia in middle-aged and older non-obese adults. OR, odd ratios; CI, confidence interval.

Taking the middle-aged and older non-obese adults without sarcopenia from these 17 studies, as the reference group, sarcopenia patients had a higher odds ratio of having MetS (OR = 2.01; 95% CI: 1.63–2.47; *p* < 0.001; *I*^2^ = 79.2%; Figure 3) using the random-effect model. This indicated that this population (with sarcopenia) was 2.01 times more likely to have MetS than middle-aged and older non-obese adults without sarcopenia. Begg’s and Egger’s tests produced *p* values of 0.669 and 0.2, respectively, suggesting the absence of publication bias.

Considering that high heterogeneity was found, sub-analyses were conducted to address the possible source of heterogeneity. Subgroup analyses that adjusted for potential confounders or not, including geographic region, MetS definition, sarcopenia measurement, cohort size (*n* ≥ 1000 versus *n* < 1000), and the method of defining non-obese versus obese, were conducted. These subgroup analyses showed that the association between MetS and sarcopenia in middle-aged and older non-obese adults was greater in studies that used unadjusted estimates, used DXA to diagnose sarcopenia, and those with cohorts of *n* ≥ 1000 (Table 3). Some results from the subgroup analyses became non-significant, which may be due the small number of studies. It is also worth noting that the overall significance of the subgroup analyses results decreased the most in studies with cohort sizes < 1000. 

Together, these sub-analyses suggested that heterogeneity could be partly explained by differences that existed (whether using adjusted data or not) from the different methods used to diagnose sarcopenia and cohort size. These factors may be important considerations to keep in mind when deriving and interpreting effect estimates from such studies in a meta-analysis.

## 4. Discussion

To the best of our knowledge, this is the first meta-analysis to investigate the association between MetS and sarcopenia in middle-aged and older non-obese adults. In summary, our results indicated that across cross-sectional data, approximately 36.45% of middle-aged and older non-obese adults with sarcopenia have MetS, while only 22.81% of this population without sarcopenia have MetS. Similar results were found by Suliga et al., in which MetS was diagnosed in 17.27% of individuals aged 37–66 years of normal weight [35]. In this meta-analysis of 13 studies with 35,581 individuals, we found a statistically significant positive OR between MetS and sarcopenia in middle-aged and older non-obese adults, indicating that the concomitant presence of MetS and sarcopenia is common in this population. 

We also found that MetS prevalence in middle-aged and older non-obese adults with or without sarcopenia in North America was higher than in Asia, which was consistent with previous studies. The association between MetS and sarcopenia showed the same regional difference. Thus, middle-aged and older non-obese adults in Asia may be more susceptible to developing MetS. However, considering that the majority of studies were conducted in Asia, more data is needed to definitively draw this conclusion. We also found that the MetS prevalence was lower in studies that only used HGS to diagnose sarcopenia, which can be explained by a lack of assessing muscle mass loss, the most important feature of sarcopenia. Individuals with sarcopenia who were diagnosed by 24-h urinary creatinine tests showed a higher MetS prevalence. As a metabolic product of creatine in muscle, creatinine levels are proportional to total muscle mass; thus, the test may have a higher sensitivity for identifying sarcopenia and MetS than other methods [27,36]. There was no apparent difference in the MetS prevalence as defined by NCEP/ATPIII or AHA/NHLBI/IDF in our analysis. This is easy to understand; both methods use the same five factors to form MetS diagnosis and differ only in the thresholds used for fasting glucose [37]. It is worth mentioning that the sarcopenia definition was extremely varied among the included studies, which might confound the association between sarcopenia and MetS. Sarcopenia defined by the aLM/BMI method, which considers height and weight together, was better for predicting disability in middle-aged and older adults than the aLM/ht^2^ method, because it considers fat as a part of the definition. Additionally, the FNIH Sarcopenia Project has recommended the definition of low muscle mass adjusted for body mass to examine sarcopenia in the general population [38]. A lower polled OR of MetS was found in our included studies with cohorts size < 1000, although there was no significant difference between the cohort size subgroups. Thus, studies with larger cohorts are required to achieve reliable results. 

### 4.1. Potential Biological Mechanisms

Our results in middle-aged and older non-obese adults were not surprising because aging is known to contribute to sarcopenia and MetS development. Skeletal muscle is a potential anti-oxidative organ during the inflammatory immune response [39]. However, the onset of decreased total lean body mass can be detected as early as the age of 45. Aging is associated with immuosenescence and accompanied by a chronic inflammatory state which leads to MetS [40]. In addition to the aging process, decreased physical activity is another cause of sarcopenia that might contribute to MetS. Some studies have shown that physical inactivity contributes to sarcopenia development [41,42]. Steffl et al. [43] conducted a systematic review and meta-analysis that confirmed the benefits of physical activity against sarcopenia development in older people. He et al. [44] performed a meta-analysis of prospective cohort studies and showed a beneficial effect of leisure time physical activity on MetS. Among these retrieved studies, seven provided adjusted ORs, and the overall effect showed a positive relationship between sarcopenia and MetS in our recruited population. Considering most adjusted variables included factors such as age, gender, smoking and alcohol consumption, the association between sarcopenia and MetS could not be simply explained by aging and physical inactivity, suggesting that other specific mechanisms may exist. As mentioned previously, the decreased myokine secretion from type IIb skeletal muscle fibers during aging cannot adequately counteract the pathological consequences of metabolic disorders that arise from adipokines secreted by adipose tissue that contribute to MetS development [21,45]. Additionally, skeletal muscle accounts for approximately half of the lean body mass of an adult individual and therefore plays an important role in insulin-stimulated glycolipid metabolism [46].

### 4.2. Directions for Further Research

There remain several questions that require further research. First, given that MetS development varies by sex, how gender influences the prevalence and effects of MetS in middle-aged and older non-obese adults with sarcopenia remains and open question. Additionally, the impact of sarcopenia on MetS in middle-aged and older obese adults is unknown. Although MetS can be seen in non-obese individuals and develops independent of obesity, obesity always contributes to MetS-associated metabolic alterations [47]. To address these issues, more studies with detailed data stratified by sex and sarcopenic obesity are needed.

### 4.3. Strengths and Limitations

Our study had multiple strengths. First, our analysis focus on the association between sarcopenia and MetS in middle-aged and older non-obese adults. We included a representative sample in which a decrease in total lean body mass began to appear. Additionally, the pooled effect estimates did not change significantly whether adjusted for potential confounders or not, indicating that our results were credible. Nevertheless, this meta-analysis also had several limitations. First, we were limited to performing this meta-analysis on observational studies. This meta-analysis mainly came from cross-sectional studies, thus, the directional causality between sarcopenia and MetS in middle-aged and older non-obese adults cannot be ascertained and requires future cohort studies for confirmation. Second, the association between sarcopenia and MetS could be influenced by the difference in adjusted factors in each study. Additionally, there were certain potential unadjusted confounding factors that might have great influence on our results. Third, the subjects of this meta-analysis were mostly Asian (and most of them Korean); better-designed studies with populations from other countries are needed. Fourth, previous studies lack data from elderly people; future studies need to focus on the relation between sarcopenia and MetS in older people. Finally, as mentioned previously, the definition of sarcopenia varies greatly among these studies, which may have confounded our results.

## Figures and Tables

**Table 1:** Characteristics of included studies.

Author, Year	Country, Setting	Study Design	Cohort Size (Female %)	Average Age, Year	Definition of Sarcopenia	Definition of MetS	Adjustments	Quality Assessment
Kang et al., 2017 [25]	Korea, community-dwelling	Cross-sectional	2574 (100%)	Sarcopenia: 61.9 ± 0.5 Control: 61.6 ± 0.3	Skeletal muscle mass measured by DXA; ASM/Wt was less than 1 standard deviation below the mean of the young reference group	AHA/NHLBI IDF/	Menopausal age, any female sex hormonal treatment, drinking status, smoking status, physical activity, chronic disease, and economic status	
Ma et al., 2016 [27]	Korea, community-dwelling	Cross-sectional	709 (53.9%)	Sarcopenia: 71.6 ± 8.0 Control: 67.7±7.0	Skeletal muscle mass evaluated by 24-h UC method; 24-h UC ≥ 12.33 mmol/day for men and ≥10.43 mmol/day for women	NCEP-ATPIII	N/A	
Han et al., 2016 [28]	United states, community-dwelling	Cross-sectional	2326 (54.4%)	Sarcopenia: 66.0 ± 12.6 Control: 51.0 ± 15.8	Skeletal muscle mass measured by DXA; ASM/BMI < 0.789 for men and <0.512 for women	NCEP-ATPIII	N/A	
Chung et al., 2016 [29]	Korea, community-dwelling	Cross-sectional	1377 (42.6%)	Sarcopenia: 62.7 ± 1.1 Control: 59.6 ± 0.3	Skeletal muscle mass measured by DXA; (ASM/Ht^2^)/Wt % < 28.9% for men and <22.4% for women	NCEP-ATPIII	Age, sex, household income, current smoking, alcohol consumption, vitamin D, hypertension and dyslipidemia	
Byeon et al., 2015 [31]	Korea, community-dwelling	Cross-sectional	5001 (N/A)	Sarcopenia: 56.4 ± 2.0 Control: 43.6 ± 0.4	Skeletal muscle mass measured by DXA; ASM/Wt % < 26.88% for men and 21.02% for women	NCEP-ATPIII	N/A	
Lee et al., 2015 [30]	Korea, community-dwelling	Cross-sectional	10,479 (65%)	Sarcopenia: 56.7 ± 16.3 Control: 47.9 ± 16.8	Skeletal muscle mass measured by DXA; ASM/Wt % < 32.2% for men and <25.5% for women	NCEP-ATPIII	N/A	
Scott et al., 2015 [26]	Korea & Australian community-dwelling	Cross-sectional	1381 (54.7%)	N/A	Skeletal muscle mass measured by DXA; ASM/BMI < 0.789 for men and <0.512 for women	NCEP-ATPIII	Age and gender	
Moon et al., 2014 [20]	Korea, community-dwelling	Cross-sectional	10,432 (56.3%)	Sarcopenia: 59.8 ± 14.3 Control: 48.3 ± 15.5	Skeletal muscle mass measured by DXA; ASM/Wt % < 26.98% for men and 21.14% for women	NCEP-ATPIII	Age, sex, region, smoking, alcohol consumption, exercise, and family income level	
Ishii et al., 2014 [18]	Japan, community-dwelling	Cross-sectional	1971 (50.4%)	Sarcopenia: 77.1 ± 5.8 Control: 72.0 ± 5.0	Skeletal muscle mass measured by BIA; SMI < 7.0 kg/m^2^ for men and <5.8 kg/m^2^ for women; muscle strength < 30 kg for men and <20 kg for women; gait speed < 1.26 m/s for each sex	NCEP-ATPIII	Age, height, weight, physical activity and food intake.	
Liu et al., 2014 [32]	Taiwan (China), community-dwelling	Cohort study, baseline data	444 (0%)	Sarcopenia: 83.1 ± 4.8 Control: 82.0 ± 4.5	Handgrip strength < 22.5 kg	NCEP-ATPIII	N/A	
Kim et al., 2013 [34]	Korea, community-dwelling	Cross-sectional	214 (80.4%)	Sarcopenia: 51.0 ± 10.6 Control: 44.2 ± 14.9	Skeletal muscle mass measured by DXA; ASM/Wt % was less than 1 standard deviation below the mean of the young reference group	NCEP-ATPIII	N/A	
Lu et al., 2012 [33]	Taiwan (China), community-dwelling	Cross-sectional	420 (72.9%)	Sarcopenia: 61.1 ± 9.6 Control: 64.4 ± 10.3	Skeletal muscle mass measured by BIA; ASM/Wt % < 37% for man and <27.6% for women	NCEP-ATPIII	Age, gender, current smoking, current drinking, vegetarian diet and physical activity	

Abbreviations: AHA/NHLBI/IDF, the American Heart Association, the National Heart, Lung, and Blood Institute, and the criteria of the International Diabetes Federation; ASM, appendicular skeletal muscle mass; BIA, bioelectric impedance analysis; BMI, body mass index; DXA, dual energy X­ray absorptiometry; MetS, metabolic syndrome; N/A, not available; NCEP-ATPIII, the National Cholesterol Education Program Adult Treatment Panel III; SMI, skeletal muscle mass index; Wt, weight.

**Table 2:** Meta-analysis results of prevalence of metabolic syndrome in middle-aged and older non-obese adults with or without sarcopenia.

Analysis	Number of Studies	Number of Participants	Meta-Analysis	Between Group *p* Value	*I*^2^
Prevalence of MetS in non-obese middle-aged and older people with sarcopenia					
			Prevalence (95% CI)		
All studies (D + L)	10	4427	0.36 (0.28–0.45)		96%
Continent				0.0364	
Asian	9	4024	0.35 (0.26–0.45)		96%
North America	1	403	0.47 (0.42–0.52)		-
Definition of MetS				0.6102	
NCEP-ATPIII	9	3870	0.37 (0.27–0.47)		97%
AHA/NHLBI/IDF	1	557	0.34 (0.30–0.38)		-
Sarcopenia measure				<0.0001	
DXA	7	3389	0.41(0.30–0.52)		96%
24 h urinary creatinine	1	403	0.47 (0.42–0.52)		-
BIA/HGS/GS	1	359	0.28 (0.24–0.33)		-
HGS	1	276	0.13 (0.10–0.18)		-
Cohort size (*n*)				0.5333	
*n* ≥ 200	6	4043	0.34 (0.25–0.44)		90%
*n* < 200	4	384	0.40 (0.24–0.59)		97%
Prevalence of MetS in non-obese middle-aged and older people without sarcopenia					
			Prevalence (95% CI)		
All studies (D + L)	10	31,154	0.23 (0.18–0.29)		99%
Continent				<0.0001	
Asian	9	30,848	0.21 (0.16–0.26)		99%
North America	1	306	0.48 (0.42–0.53)		-
Definition of MetS				0.7922	
NCEP-ATPIII	9	31,154	0.23 (0.18–0.29)		99%
AHA/NHLBI/IDF	1	2071	0.23 (0.22–0.25)		-
Sarcopenia measure				<0.0001	
DXA	7	29,068	0.20 (0.16–0.25)		99%
24-h urine	1	306	0.48 (0.42–0.53)		-
BIA/HGS/GS	1	1612	0.38 (0.36–0.40)		-
HGS	1	168	0.12 (0.08–0.18)		-
Cohort size (*n*)				0.0081	
*n* ≥ 200	8	30,800	0.27 (0.21–0.34)		99%
*n* < 200	2	354	0.08 (0.03–0.19)		84%

Notes: MetS, metabolic syndrome; NCEP-ATPIII, National Cholesterol Education Program Adult Treatment Panel III; AHA/NHLBI/IDF, American Heart Association, the National Heart, Lung, and Blood Institute, and the International Diabetes Federation; DXA, dual-energy X-ray absorptiometry; BIA, bioelectrical impedance analysis; HGS, hand grip strength; GS, gait speed.

**Table 3:** Pooled ORs and 95% CIs of the associations between MetS and sarcopenia.

			Heterogeneity	
Subgroups	Number of Studies	OR (95% CI)	*I*^2^ (%)	*P*_heterogeneit_
All studies	13	2.01 (1.63–2.47)	79.2	<0.001
Adjustment				
Adjusted	7	1.85 (1.48–2.32)	50.4	0.060
Unadjusted	6	2.16 (1.54–3.03)	86.0	<0.001
Continent				
Asian	11	2.12 (1.69–2.65)	74.8	<0.001
North America	1	1.61 (1.31–1.97)	-	-
Asian & Oceania	1	1.64 (1.12–2.41)	-	-
Definition of MetS				
NCEP-ATPIII	12	2.01 (1.59–2.53)	80.4	<0.001
AHA/NHLBI/IDF	1	1.97 (1.51–2.57)	-	-
Sarcopenia measurement				
DXA	8	2.41 (1.98–2.94)	63.1	0.008
BIA/HGS/GS	2	1.44 (0.72–2.87)	74.6	0.047
24 h urinary creatinine	1	1.61 (1.31–1.97)	-	-
BIA	1	1.98 (1.25–3.15)	-	-
HGS	1	1.08 (0.61–1.92)	-	-
Cohort size				
*n* ≥ 1000	7	2.41 (1.96–2.97)	68.4	0.004
*n* < 1000	6	1.53 (1.22–1.93)	32.8	0.190
Non-obese evaluation				
Divided by BMI	11	1.92 (1.53–.41)	78.9	<0.001
Adjustment for BMI	2	2.01 (1.63–2.47)	89.1	<0.001

Notes: MetS, metabolic syndrome; NCEP-ATPIII, National Cholesterol Education Program Adult Treatment Panel III; AHA/NHLBI/IDF, American Heart Association, the National Heart, Lung, and Blood Institute, and the International Diabetes Federation; DXA, dual-energy X-ray absorptiometry; BIA, bioelectrical impedance analysis; HGS, hand grip strength; GS, gait speed; BMI, body mass index.

## Supplementary Materials

The following are available online at http://www.mdpi.com/2072-6643/10/3/364/s1; Table S1: The Preferred Reporting Items for Systematic Reviews and Meta-Analysis (PRISMA) statement.
